# Clouds over IMD? Perspectives for inherited metabolic diseases in adults from a retrospective cohort study in two Swiss adult metabolic clinics

**DOI:** 10.1186/s13023-020-01471-z

**Published:** 2020-08-18

**Authors:** Karim Gariani, Marina Nascimento, Andrea Superti-Furga, Christel Tran

**Affiliations:** 1 Division of Endocrinology, Diabetes, Nutrition and Therapeutic Patient Education, Geneva University Hospitals, Geneva, Switzerland; 2 University of Lausanne Faculty of Biology and Medicine, Lausanne, Switzerland; 3 Center for Molecular Diseases, Division of Genetic Medicine, Lausanne University Hospital, Beaumont-02/248, 1011 Lausanne, Switzerland

**Keywords:** Inherited metabolic diseases, Adult metabolic clinic, Treatment, Complications

## Abstract

**Background:**

Inherited metabolic diseases (IMD) are complex medical conditions. Thanks to improvements in diagnosis and treatment, a growing number of pediatric IMD patients reach adulthood. Thus, clinical care of adults with IMD has emerged as a new and challenging reality. This purpose of this study of adults with IMD in an adult metabolic clinic at two academic hospitals (Lausanne and Geneva) was to help inform decisions on the future organization of health care for this group of patients.

**Methods:**

All adult patients with a biochemical and/or genetic diagnosis of IMD followed at the clinics were included in the study. Electronic patient records were reviewed for clinical features, diagnostic studies, treatment and long-term outcome. Data of undiagnosed patients referred for suspected IMD were analyzed separately.

**Results:**

126 patients were included in the study. The most prevalent group of diseases was small molecules disorders with 82 (65%) patients, followed by energy defects disorders with 29 (23%) patients and complex molecules disorders with 15 (12%) patients. Overall, 64% of patients were diagnosed before, and 36% after the age 16 years. Among the 126 cases, 51% suffered from medical complications. 79% of the patients were receiving a specific treatment for their disease. Among the 138 undiagnosed patients referred for suspicion of IMD, investigations lead to a genetic diagnosis in 24 (17%) patients. 19 had confirmation of an IMD, 5 were found to have another genetic condition.

**Conclusions:**

This retrospective study reveals significant features of adult IMD cohort. The disorders are heterogeneous, and there is no one-size-fits-all approach – treatment must be tailored to fit each specific disorder in each individual patient. Even patients who are followed at the dedicated clinic are not protected from metabolic decompensations and/or chronic organ-specific complications. While it is commonly assumed that patients with IMD are more stable once they become adults, our data show that the diseases continue to exact a lifelong toll. A coordinated monitoring of target organs by a multidisciplinary team is needed. To ensure that the success in diagnosis and treatment of individuals with IMD is sustained, there is a clear requirement for adequately staffed adult IMD clinics.

## Introduction

Inherited metabolic diseases (IMD) are genetic disorders resulting from an enzyme defect and/or transport proteins in metabolic pathways affecting proteins, fat, carbohydrate metabolism or impaired organelle function. IMD present as complex medical conditions involving multiple organ systems [1]. The most recent nosology of IMD includes well over 1000 disorders [2]. Even though they are individually rare, their collective prevalence is estimated today at greater than 1:800 individuals [3]. Improvements in screening programs, disease awareness, diagnostic tests and therapeutic interventions in IMD have led to increasing patient survival and better prognosis but also to the detection of milder or late-onset forms that present in adulthood [4–7]. A recent survey of the metabolic European reference network showed that 50% of patients with IMD are adults (Scientific report, Board annual meeting 2018, MetabERN).

In the past, IMD medicine has been considered as a pediatric discipline and no formal training has been offered for adult medicine. As a result, many of these patients are still under the care of their pediatricians as young adults [8, 9]. As with other chronic illnesses, numerous obstacles are known (i.e. lack of support system, unfamiliarity with IMD, poor adherence to treatment) which may interfere with the transition process [10, 11]. Little is known on long-term outcomes of childhood IMD as many of the “first survivors” are only now approaching middle age. Exploring the long-term outcomes of these patients is, nonetheless, important from the viewpoint of developing the optimal treatment strategy over a lifetime [12]. In order to meet this growing demand, programs with a higher level of formal training in adult IMD are underway in several countries [13] leading to adult metabolic physicians specializing in this new and expanding medical discipline [14]. However, according to a recent survey, most of the centers following adult patients with IMD felt the current state of available education and training in this field were insufficient [15].

In our country, an adult metabolic clinic was established in 2013 at the Centre for Molecular Diseases of the University Hospital of Lausanne and was extended in 2015 by an affiliation with the Division of Endocrinology, Diabetology, Hypertension and Nutrition of the University Hospitals of Geneva. This study was intended to establish a database of the current health of adult patients with IMD in order to better understand their clinical course and to obtain a basis upon which to organize the future health care provision for these patients.

## Materials and methods

This study was approved by the Swiss Ethics Committees on research involving humans (Approval # 2017–02328) and was registered at the U. S Clinical Trials Registry as NCT03534752. All patients with a biochemical and/or genetic diagnosis of IMD aged ≥16 years and followed at the adult metabolic clinic from October 1st 2013 to December 31st 2017 were included. Patient were divided into three groups according to the pathophysiology and using a simplified classification of IMD: complex molecule disorders, small molecule (intoxication) disorders and energy defects disorders [16]. Electronic and paper patient charts from the Division of Genetic Medicine (Lausanne University Hospital) and the Division of Endocrinology, Diabetology, Hypertension and Nutrition (Geneva University Hospitals) were reviewed for clinical features, biochemical investigations, molecular genetic testing, treatment, number of hospitalizations, complications of the disease and long-term outcome. Dietary measures, special blend of amino acids formula, vitamins, cofactors and any orphan drug were considered as specific treatment for IMD. Only complications occurring during the time of analysis (from October 1st 2013 to December 31st 2017) and related to IMD were included in the analysis.

A second analysis included all the patients referred from October 1st 2013 to December 31st 2017 to the Adult Metabolic Clinic for suspicion of IMD and for whom further investigation had confirmed an IMD disease or not. We focused [1] on the reason for their referral (i.e. positive family history, suggestive symptoms or biochemical abnormalities) and [2] the frequency of confirmed IMD diagnosis, non-IMD diagnosis (other cause) and unsolved cases. All data were entered in an Excel file and were analyzed by research team members (MN, KG and CT).

### Statistical analysis

All data were analyzed using Excel statistical functions for Office 365 for Mac and for Windows 2016. Frequency was calculated for classification of patients according to the metabolic subtype. Prevalence was reported as percentage of the total group or subgroup of patients depending on the studied outcome. Data for age at the time of analysis were presented as mean ± SD.

## Results

### General

126 adult patients with IMD including 64 men (51%) and 62 women (49%) were enrolled in the study. One hundred (79%) of the patients had transitioned from the pediatric metabolic clinics whereas 26 (21%) were referred from another specialty. The mean age (in years) at the time of analysis for patients in the three subgroups (complex molecules, small molecules and energy defects) was 36.5 ± 14.9, 29.6 ± 11.0 and 33.6 ± 15.4, respectively. An overview of the main results is summarized in Table 1.

**Table 1:** Results by diagnosis subgroups

		Complex molecules disorders	Small molecules disorders	Energy defects disorders	Total	Percentage (%)
Number of patients		15 (12%)	82 (65%)	29 (23%)	**126**	
Gender	Male	10	35	19	64	51
	Female	5	47	10	62	49
Age at diagnosis	Infantile	0	38	2	40	32
	1 to 15 years	9	23	8	40	32
	Over 16 years	6	21	19	46	37
Transition from pediatric metabolic clinic		11	70	19	100	79
New patients		4	12	10	26	21
Specific treatment		9	67	23	99	79
Identified pathogenic variant		10	29	24	63	50
Complications related to the disease		8	39	17	64	51
Hospitalized at least once		5	26	14	45	36
Hospitalized due to a metabolic origin		6	15	12	33	73
Lost to follow-up		1	11	3	15	12
Deceased		1	1	0	2	2

### Disease frequencies

The most prevalent group of diseases was small molecules disorders with 82 (65%) patients, followed by energy defect disorders with 29 (23%) patients and complex molecules disorders with 15 (12%) patients. Disorders of phenylalanine metabolism were the most represented disease with 16 patients having classical phenylketonuria (PKU) and seven mild hyperphenylalaninemia (supplementary Table 1). The most prevalent group in complex molecule disorders was sphingolipidosis (lysosomal) with Gaucher and Niemann-Pick type B diseases representing eight patients in total. Patients with Fabry disease were not included in this study as they were followed by a specific and separate clinic. The most prevalent diseases in the energy defect disorders were Chronic Progressive External Ophthalmoplegia (CPEO) and Mitochondrial Encephalopathy with Lactic Acidosis and Stroke-like episodes (MELAS) with a total of 10 patients.

### Age group at diagnosis and molecular analysis

Age group at diagnosis is reported in Table 1. Overall the distribution of diagnosis by age was roughly equivalent among the three age groups (infantile, 1 to 15 years, over 16 years). In the subgroup analysis, the majority of patients with small molecule disorders were diagnosed during infancy (46%), while patients in the energy group were diagnosed after the age of 16 years (66%). Age at diagnosis in complex molecule disorders was widely distributed between the different age groups. Factors associated with diagnosis in adulthood were: [1] *true late-onset form of the disease*. Some gene variants are known to be associated with a milder, later onset phenotype (ex. the c.-32-13 T > G variant found in one of our patient is reported in approx. 80–90% of late-onset Pompe disease [17, 2] *Delayed diagnosis of infantile forms*. Some of the adult patients diagnosed lately had their first symptoms in infancy (i.e. splenomegaly in Niemann-Pick B), and [3] *genotypes known to be associated with variable clinical presentation*, such as in mitochondrial disease. The diagnosis was based solely on biochemical results in 50% of the cohort, while molecular genetic confirmation was obtained in the other half. Of the 63 patients with genetic confirmation of the pathogenic mutation, 29 (46%) had small molecule, 24 (38%) energy defect and 10 (16%) complex molecule disorders.

### Disease-related complications

During the study period (4 years, 2 months), 64 (51%) of the 126 patients experienced disease-related complications. The number of complications by subgroup and their characteristics are summarized in Table 2. The majority of the complications were due to worsening of pre-existing complications (69%) followed by acute metabolic decompensation (19%), and finally, newly detected complications (13%). Among the 64 patients, 54 (84%) of them were receiving a specific treatment. In total, 45 (36%) patients had at least one hospitalization and for 33 (73%) of them it was for complications related to the IMD (i.e. acute metabolic decompensation, cardiac decompensation, epilepsy, leukoencephalopathy).

**Table 2:** Characteristics of the complications related to the underlying inherited metabolic diseases arising during the time of the study (total number of patients, *N* = 64)

**Acute Metabolic decompensation (*N*** **= 12)**			
Group	Disease	Description	Number (N)
2	Urea cycle disorders	Hyperammoniemia	4
2	Leucinosis (Maple syrup urine disease)	Encephalopathy with hyperleucinosis and Hyperammoniemia	1
2	Fructose-1,6-diphosphatase deficiency	Hypoglycemia	1
2	Classical homocystinuria	Hyperhomocysteinemia	4
3	HI/HA syndrome	Hypoglycemia and hyperammoniemia	1
3	Carnitine palmitoyltransferase 2	Rhabdomyolysis	1
**Novel complications (*N*** **= 8)**			
Group	Disease	Description	
1	Pompe disease	Dysphagia	1
1	X-ALD	Myeloneuropathy	1
2	Arginosuccinic aciduria	Dilated left ventricule	1
2	Fructose-1,6-diphosphatase deficiency	Hepatomegaly	1
2	Galactosemia	Osteopenia	1
2	Biotinidase deficiency	Distal motor involvement	1
3	Kearns-Sayre syndrome	Heart bifascicular block	1
3	Leigh syndrome	Epilepsy	1
**Worsening of pre-existing complication (*N*** **= 44)**			
Group	Disease	Description	
1	Gaucher disease type I	Fatigue	1
1	Niemann-Pick type B	Restrictive lung disease, liver cirrhosis, portal hypertension, splenomegaly	4
1	Mucopolysaccharidosis type IVA	Tracheal stenosis	1
1	Mucopolysaccharidosis type II	Repetitive urinary tract and respiratory infection	1
2	Hereditary fructose intolerance	Liver steatosis	1
2	Vitamin B12 unresponsive MMA	Dystonia, kidney failure post transplant	2
2	Acute intermittent porphyria	Depression	1
2	Wilson disease	Dystonia, cirrhosis, depression	4
2	Mild hyperphenylalaninemia	Behavioral disorders	
2	Classical galactosemi	Primary ovary insufficiency, optic atrophy, anxiety, osteopenia	5
2	Phenylketonuria	Epilepsy	2
2	Classic homocystinuria	Kidney failure, short bowel syndrome, osteoporosis	4
2	OAT deficiency	Gyrate atrophy	1
2	Leucinosis	Spastic diplegia	1
2	Cobalamin A deficiency	Kidney failure	1
2	Cobalamin C deficiency	Left ventricular dysfunction, mild mental retardation, bilateral retinopathy	2
2	Lesh-Nyhan syndrome	Gastro-intestinal intolerance	1
3	CPEO	Balance problems, myopathy, palpebral ptosis	4
3	MELAS	Cardiomyopathy, diabetes, kidney failure	3
3	Mitochondrial complex III deficiency	Fanconi syndrome	1
3	Ovario leucodystrophy related to AARS2 mutation	Cognitive decline, myopathy	1
3	Glycogen storage disease type 3	Myalgia	2
3	KSS	Diabetes	1

*Abbreviations*: *CPEO* chronic progressive external ophthalmoplegia, *HI/HA* hyperinsulinism/hyperammoniemia, *KSS* Kearns-Sayre Syndrome, *MELAS* mitochondrial encephalopathy with lactic acidosis and stroke-like episodes, *MMA* methylmalonic acidemia, *OAT* ornithine aminotransferase, *X-ALD* X-linked adrenoleucodystrophy

Group: 1) Complex molecules disorders, 2) Small molecules disorders, 3) Energy defect disorders

### Lost to follow-up and death

During the time of the study, 15 patients were lost to follow-up. Five of them had been seen only once in clinic and none of these patients had regular follow-up primarily. Two of them relocated to another area with an orderly transition to another adult metabolic clinic. For two of them, it was due to lack of coordination between the different specialists in a multidisciplinary context. Finally, six patients freely decided to discontinue regular follow-up in our clinic. Factors associated with loss of follow-up were poor adherence to treatment, independence from parental control after transition, integration in working life with limited availability, and living at longer distance from our clinic. Two patients passed away. One due to the natural history of the disease (Lesch-Nyhan syndrome) and one whose death outside the hospital was due to a possible cardiovascular event (Niemann-Pick B).

### New adult referrals

One hundred and thirty-eight (62 men and 76 women) were referred to the metabolic clinic for a suspicion of IMD. 133 patients were referred for symptoms and 5 for a positive family history. The ones in which an IMD was confirmed were included in the statistics of the general results. Of the 138 referrals for IMD suspicion, a genetic diagnosis was reached in 24 patients (17%): 19 with a genuine IMD and 5 with a non-IMD genetic disease. Three patients were found with a possibly disease-related polymorphisms but not considered as genetic disease (Table 3). Most of the diagnoses were made in patients who had positive family history or neurological symptoms with clear neurological findings (i.e. abnormal electroneuromyography, elevated creatine kinase (CK), signs of myopathy). Rare genetic but non-IMD diseases were found using whole exome sequencing. Every new diagnosis has resulted in genetic counseling. Nevertheless, a relatively low number of related individuals has been identified. Two main reasons were identified: first, adult patients often have less or no contact with their families; and second, for adult individuals the clinical observation is often sufficient to rule out disease. Investigations were inconclusive for all the patients referred for chronic fatigue syndrome, myalgia (with no elevation of CK), chronic/joint pain but normal clinical examination (data not shown).

**Table 3:** Undiagnosed patients: reasons for referral and diagnosis

ID	Reason for referral	IEM diagnosis	OMIM#
1	Myopathy, ptosis	Mitochondrial disease (CPEO)	609,286
2	Epilepsy, ophthalmoplegia, ptosis	Mitochondriopathy disease (ARPEO)	258,450
3	Myopathy, arthralgia, cognitive delay	Myoadenylate deaminase deficiency	615,511
4	Myopathy, ovary insufficiency, cognitive decline, palpebral ptosis	Mitochondrial disease (ovario leucodystrophy related to *AARS2* variant)	615,889
5	Myopathy, respiratory insufficiency	Heterozygous for Pompe late-onset disease (2nd variant not found)	232,300
6	Myopathy, respiratory insufficiency	Pompe late-onset disease	232,300
7	Myopathy, ophthalmoplegia, ptosis	Mitochondrial disease (CPEO)	609,286
8	Pulmonary embolism	Homocystinuria due to CBS deficiency	236,200
9	Intestinal ischemic thrombosis, pulmonary embolism	Homocystinuria due to CBS deficiency	236,200
10	Myopathy, ptosis	Mitochondrial disease (KSS)	530,000
11	Biochemical hypermethioninemia, cognitive delay	Methionine adenosyltransferase I/III deficiency	250,850
12	Progressive myelopathy	X-linked AMN	300,100
13	Splenomegaly, liver cirrhosis, bone lesions	Niemann-Pick type B	607,616
14	Hypoglycemia, hyperammoniemia	HI/HA syndrome	606,762
15	Positive FHx for OTC	OTC deficiency	311,250
16	Positive FHx for CBS deficiency	Homocystinuria due to CBS deficiency	236,200
17	Positive FHx for CBS deficiency	Homocystinuria due to CBS deficiency	236,200
18	Positive FHx for CBS deficiency	Homocystinuria due to CBS deficiency	236,200
19	Positive FHx for CBS deficiency	Homocystinuria due to CBS deficiency	236,200
**ID**	**Reason for referral**	**Other Genetic diagnosis**	
20	Spastic paraparesis, cognitive delay	Spastic paraparesis related to *SPG11* variant	604,360
21	Cerebral calcification, leukoencephalopathy	Nasu-Hakola syndrome (PLOSL)	618,193
22	Peripheric weakness, ataxia, ophthalmoplegia	Autosomal dominant spinocerebellar ataxia 5	600,224
23	Familial neuropathy, muscle weakness	Charcot-Marie Tooth related to *LRSAM1* variant	614,436
24	Cognitive decline, peripheric neuropathy, polyglucosan bodies	Charcot-Marie Tooth related to *HARS1* variant	616,625
**ID**	**Reason for referral**	**Possibly related polymorphisms**	
25	Mild elevation of homocysteine	MTHFR polymorphism (compound heterozygous)	NA
26	Mild elevation of homocysteine, venous thrombosis	MTHFR polymorphism (compound heterozygous)	NA
27	Recurrent pregnancy loss	MTHFR polymorphism (compound heterozygous)	NA

Abbreviations: ARPEO: autosomal recessive progressive external ophalmoplegia; AMN, adrenomyeloneuropathy; CPEO: chronic progressive external ophthalmoplegia; CBS: cystathionine-β synthase deficiency; FHx: family history; HI/HA, hyperinsulinism/hyperammoniemia; ID; identity number, KSS: Kearns-Sayre Syndrome; MTHFR, methylenetetrahydrofolate reductase; NA: not applicable; OMIM: online mendelian inheritance in man; OTC, ornithine transcarbamylase; PLOSL, polycystic lipomembranous osteodysplasia with sclerosing leukoencephalopathy; X-ALD, X-linked adrenoleucodystrophy

## Discussion

We undertook this study to obtain a global appreciation of the current state of adult IMD medicine in the French-speaking part of Switzerland. Some numerical results, as presented below, were in line with those obtained in other countries and centres. However, we were struck by what looked like a high burden of disease in our patients, and this is the aspect we would like to emphasize.

Which are the limitations of our study? Firstly, patients with Fabry disease were not included in this study as they were followed at a parallel dedicated clinic. However, previous studies showed that Fabry disease was one of the most prevalent IMD in adults with diagnosis almost exclusively in adulthood [12, 18]. This may affect the interpretation of the disease frequencies and age group at diagnosis in our cohort and its comparison with previous studies. Secondly, some patients with IMD in the French-speaking Switzerland may be followed by other specialists or centres and therefore were not included in our results; this may be particularly true for individuals with less severe forms. Thirdly, classification of IMD is variable according to the textbooks and literature. We based our criteria on a reference textbook for inherited metabolic disease in adult [19], the Vademecum Metabolicum [20] and the Saudubray classification [16, 21] but are aware that it may differ from other classifications such as the MetabERN diseases group (European Reference Network for Inherited Metabolic Disorders) [22]. Fourthly, not all IMD are represented in our cohort (i.e. congenital disorders of glycosylation, some urea cycle disorders, and few subtypes of mucopolysaccharidosis, glycogenosis and β-oxidation defect) which also significantly biases the interpretation of the results. For these reasons, we do not suggest that our figures are necessarily applicable to other settings and other countries. In spite of these shortcomings, we feel that our study offers some insights that may be worthy of reflection.

### Comparison of the size of our clinic and its disease distribution to other clinics around the world

In 2015, The SSIEM Adult Metabolic Physician Group completed a survey of 15 centers worldwide with a total number of 6182 adult patients [18]. The number of patients with confirmed metabolic diagnosis varied widely between centers (from 10 to 1940). London had the highest number of patients (1940), followed by Vancouver (795) and Amsterdam (600). At that time, no Swiss Center was included. Retrospectively our number of patients was close to the number of the Reference Centers for Inherited Metabolic Diseases in Lille, France (126), in Udine, Italy (117) and in Hamburg, Germany (155). Considering that the French-speaking part of Switzerland and the city of Hamburg both had a population of nearly 2 million in 2018, the rate of attendance is similar between these two clinics, confirming the plausibility of our data. As expected, the most frequent disorder was PKU (including hyperphenylalaninemia), representing 23 (18.3%) cases. This frequency is close to the Report of the SSIEM adult Group (20.6%) and a study from Perez-Lopez et al. (21.6%) where 500 cases of adult patients with IMD from Reference Centers in Spain were reviewed [12]. The high prevalence of PKU may also be due to the fact that it has been the first disorder included in newborn screening programs [23]. The prevalence of the other IMD in this study is comparable to previous studies, notably with amino acid (protein) metabolism disorders being the most prevalent group [18]. The LSD group was underrepresented as our cohort did not include Fabry patients.

### IMD diagnosed in adulthood account for more than 30% of all diagnoses

Thirty-seven % of the patients were diagnosed after 16 years of age. This observation was close to the SSIEM adult group report (45.7%) [18] and included mainly patients from the energy defect disease group and storage disorders. Adult-onset is frequent in mitochondrial disease where individual level of heteroplasmy and variable environment can trigger the disease at any age. In contrast, the majority of the patients in the small molecule disorders group were diagnosed in the infantile period which is likely explained by newborn screening and the frequent onset of symptoms in the neonatal period. Of note, many of our historical patients had been diagnosed solely on biochemical grounds; with the advent of quasi-universal application of next-generation sequencing techniques, the recognition of variant forms with juvenile or adult onset will increase significantly [24, 25]. Here, it must be remarked that many of these “adult” diagnoses were made because of astute observations or intuitions of individual physicians; we suspect that a large number of individuals still go unrecognized. Awareness of the indication for and the availability of genetic testing remains fragmentary among physicians caring for adult patients.

### More than 50% of patients present with medical complications

More than half of the patients developed medical complications during the time of the study. Specific IMD, particularly those involving energy deficiency (mitochondrial diseases) and storage disorders, can present with dysfunction of different systems, contributing to complications. While we did not formally measured attendance and compliance, most of the patients attended the clinic regularly (as shown by the lost to follow-up data). Some of the patients, mainly from the small molecule disorder group, were susceptible to acute metabolic decompensation, which required urgent hospital admission for correction of the metabolic derangement. Reasons for decompensation were in line with known triggers such as infection, non-adherence to treatment, or prolonged exercise [26]. Unfortunately, we observed several complications in patients for whom only conservative treatment is available with no disease-specific alternatives such as enzyme replacement therapy or other.

### The challenge continues after transition from pediatric to adult care

The transition process has been effective for the majority of patients in our cohort however a minority of patients has been lost to follow-up thereafter, highlighting that this period is fragile and needs careful attention such as described previously for other genetic and/or endocrine conditions [11, 27, 28]. In summary, the overall pattern emerging from these observations is the following: patients diagnosed in the neonatal or paediatric period benefit from improved therapy today as compared to 50 years ago and thus more patients reach adolescence and adulthood; however these patients must go through a transition process that paves the way to adulthood and its new challenges. In addition, there is a growing number of patients who are diagnosed with an IMD in adult age. As adults, all these patients may enjoy periods of stability but decompensations are possible at any time and late-onset complications appear as the patients age and the chronic genetic disease continues to exact its toll.

### Ongoing efforts to coordinate diagnosis and care of individuals with IMD

Newborn screening for various IMD has already existed in Switzerland for 50 years [29]; and in 2017, Switzerland was considered to have the third best standard of healthcare in the world [30]. Despite this remarkable track record, the observations above indicate that there is much room for improvement. Specific programmes for rare diseases have been or are being implemented in several European countries. Most of these programmes include a specific section on IMD. In Switzerland, The National Coordination for Rare Diseases (Kosek) is mandated with the establishment of networks to improve the care of individuals affected by rare diseases. One of the pilot groups within the Kosek is focused on IMD. One of the requisites of the networking progress is to make sure that dedicated centers meet high standards of organization and accountability [31]. Ultimately, national recognition should enable reference centers to integrate European reference networks [22]. Nevertheless*,* we would like to suggest that national coordination will not work unless the individual centers are improved and strengthened.

## Conclusions

In the twenty-first century, we welcome the fact that improved diagnosis and treatment are making IMD mortality a relic of the past, giving way to a growing adult population. However these advances also bring challenges for medicine and society. In spite of the limitations discussed above, this retrospective study may give some indications on the outcome of these patients and indicate where the health system might be improved further: [1] adults with IMD are clearly an emerging population in Switzerland and may soon outnumber the pediatric population. Both children and adults with IMD require specific management and expertise from the health care providers, but the requirements are different and the setting is different (pediatric clinics vs. adult clinics), [2] as more than half of our patients developed organ-specific complications, a multidisciplinary team and disease-specific personalized health plans are necessary to monitor target organs and [3] as no patient is protected from metabolic decompensations, IMD clinics must be connected to tertiary hospitals offering intensive care units as well as availability of specific drugs. Hence the need for coordination of the management of these rare diseases at the national, and ideally international, level.

## Supplementary information

**Additional file 1: Supplementary Table 1.** Diagnosis of inherited metabolic diseases (IMD) by frequency.

## Data Availability

Data and materials are available upon request to the corresponding author (CT).

## Abbreviations

CPEO: Chronic progressive external ophthalmoplegia
CK: Creatine kinase
IMD: Inherited metabolic diseases
LSD: Lysosomal storage disorders
MELAS: Mitochondrial encephalopathy with lactic acidosis and stroke-like episodes
PKU: Phenylketonuria
SSIEM: Society for the study of inborn errors of metabolism
