# Supplementary material for: Clouds over IMD? Perspectives for inherited metabolic diseases in adults from a retrospective cohort study in two Swiss adult metabolic clinics
Source: Orphanet J Rare Dis. 2020 Aug 18;15:210. doi: 10.1186/s13023-020-01471-z (PMC7433045; doi:10.1186/s13023-020-01471-z)
Supplement: Supplementary file 1 — Additional file 1: Supplementary Table 1. Diagnosis of inherited metabolic diseases (IMD) by frequency. [file 13023_2020_1471_MOESM1_ESM.docx]

| **Supplementary table 1.** Diagnosis of inherited metabolic diseases (IMD) by frequency | | |
| --- | --- | --- |
| **IMD group** | **Number of patients (n)** | **Percentage (%)** |
| **COMPLEX MOLECULES DISORDERS** |  |  |
| **Lysosomal Storage disorders** |  |  |
| Gaucher disease type I | 4 | 3.2 |
| Niemann-Pick type B | 4 | 3.2 |
| Pompe disease (type II GSD with acid maltase deficiency) | 2 | 1.6 |
| Mucopolysaccharidosis type II (Hunter) | 1 | 0.8 |
| Mucopolysaccharidosis type IVA (Morquio syndrome) | 1 | 0.8 |
| **Peroxisomal Disorders** |  |  |
| X-linked adrenoleucodystrophy/X-AMN | 2 | 1.6 |
| **Bile acid synthesis Defects** |  |  |
| Cerebrotendinous xanthomatosis | 1 | 0.8 |
| **SMALL MOLECULES DISORDERS** |  |  |
| **Disorders of protein metabolism** |  |  |
| Classical phenylketonuria | 16 | 12.8 |
| Homocystinuria due to CBS deficiency | 10 | 7.9 |
| Mild hyperphenylalaninemia | 7 | 5.6 |
| Ornithine transcarbamylase deficiency | 6 | 4.8 |
| Leucinosis (Maple syrup urine disease) | 3 | 2.4 |
| Propionic acidemia | 2 | 1.6 |
| 2-hydroxyglutaric aciduria | 1 | 0.8 |
| Argininosuccinic aciduria | 1 | 0.8 |
| Methionine adenosyltransferase I/III deficiency | 1 | 0.8 |
| OAT deficiency (gyrate atrophy) | 1 | 0.8 |
| **Disorders of carbohydrate metabolism** |  |  |
| Classical galactosemia | 6 | 4.8 |
| Duarte variant galactosemia | 2 | 1.6 |
| Hereditary fructose intolerance | 2 | 1.6 |
| Fructose-1,6-diphosphatase deficiency | 2 | 1.6 |
| **Vitamin Metabolism disorders** |  |  |
| Methylmalonic acidemia with homocystinuria type cblC | 4 | 3.2 |
| Biotinidase deficiency | 4 | 3.2 |
| B12 unresponsive methylmalonic acidemia | 2 | 1.6 |
| B12 responsive methylmalonic acidemia type cblA | 1 | 0.8 |
| Partial biotinidase deficiency | 1 | 0.8 |
| Imerslund-Gräsbeck syndrome | 1 | 0.8 |
| Urban-Rogers-Meyer syndrome | 1 | 0.8 |
| **Mineral and metal metabolism disorders** |  |  |
| Wilson disease | 5 | 4.0 |
| **Porphyrins and haem metabolism** |  |  |
| Acute intermittent porphyria | 2 | 1.6 |
| **Disorders of purine/pyrimidine metabolism** |  |  |
| Lesch-Nyhan syndrome | 1 | 0.80 |
| **ENERGY DEFECT DISORDERS** |  |  |
| CPEO | 5 | 4.0 |
| MELAS | 5 | 4.0 |
| Kearns-Sayre syndrome (KSS) | 2 | 1.6 |
| MERRF | 1 | 0.8 |
| Syndrome de Leigh | 1 | 0.8 |
| ARPEO | 1 | 0.8 |
| Mitochondrial complex III deficiency | 1 | 0.8 |
| Ovario leucodystrophy related to *AARS2* mutation | 1 | 0.8 |
| Glycogen storage disease type 1A | 1 | 0.8 |
| Glycogen storage disease type 3 | 2 | 1.6 |
| CPT2 | 2 | 1.6 |
| Carnitine transporter deficiency | 1 | 0.8 |
| MCAD deficiency | 1 | 0.8 |
| VLCAD deficiency | 1 | 0.8 |
| β-ketothiolase deficiency | 1 | 0.8 |
| X-linked creatine transporter defect | 1 | 0.8 |
| GLUT1 deficiency syndrome | 1 | 0.8 |
| HI/HA syndrome | 1 | 0.8 |
| **Total** | **126** | 100 |

| Abbreviations: AMN: adrenomyeloneuropathy; ARPEO: autosomal recessive progressive external ophalmoplegia; cbl: cobalamin; CPT2: carnitine palmitoyltransferase 2; CBS: cystathionine-β synthase; CPEO: chronic progressive external ophtalmoplegia ; GLUT : glucose transporter ; GSD: glycogen storage disorder; HI/HA : hyperinsulinism/Hyperammoniemia ; IMD: inherited metabolic diseases; MCAD : medium-chain acyl-CoA-dehydrogenase ; OAT: ornithine aminotransferase; RYR1 : ryanodine receptor 1; VLCAD : very long-chain acyl-CoA dehydrogenase |
| --- |
